# Eleventh Annual Meeting of the Miss. Val. Ass. of Dental Surgery

**Published:** 1855-04

**Authors:** 


### Proceedings of Societies.

---

ELEVENTH ANNUAL MEETING OF THE MISSISSIPPI VALLEY ASSOCIATION OF DENTAL SURGERY.

Society met pursuant to adjournment at 10 o'clock, Wednesday, February, 21, 1855, in the lecture room of the Ohio College of Dental Surgery. The President, Dr. Goddard in the chair.

Present, Drs. Taft, Taylor, Bonsal, Ulrey, Talbert, Watt, Smith, Webster, and Leslie.

On motion, Drs. Ulrey and Watt were appointed on the Executive Committee, in place of absent members. After an absence, this Committee presented the following report, which was adopted. The Executive Committee respectfully submit the following report, as the order of business.

I. Report of the Examining Committee.

II. " of Officers.

III. " of Standing Committees.

IV. Addresses and Reading of Essays.

V. Miscellaneous Business.

1st. What is the best means to allay the sensibility in teeth in which the nerves are not actually exposed.

2d. Is any non-conductor to be used previous to filling, if so, what is best ?

3d. How should teeth be treated in case the nerves are exposed.

4th. What is the best method of destroying the pulp of a tooth.

5th. What is the best material for filling teeth, and how should it be prepared.

6th. Has Risodontropy borne the test of experience.

7th. What is the best material for hard casts.

8th. Do either Allen's or Hunter's continuous gums, bear the test of mastication as well as teeth on ordinary plate or block work.

9th. What is the experience of the profession in regard to block tin, india rubber, or gutta percha bases for either temporary or permanent sets of teeth, and what is the best method of manipulation in their preparation.

10th. What is the experience of the profession in the use of chloroform and ether, in regard to mental hallucinations.

11th. How does the material known as Hill's stopping, answer as a temporary filling, and is it at all affected by the fluids of the mouth.

12th. What is the best method of inserting plates by air pressure.

A .S. TALBERT.
GEORGE WATT.
J. P. ULREY.

The Examining Committee, presented the name of W. H. Howe, D.D.S., of Cincinnati, for membership. On balloting, Dr. Howe was declared unanimously elected.

The Treasurer, presented the following report which was accepted.

*To the M. V. A. of Dental Surgery.*

GENTLEMEN:—Your Treasurer would report the following as the state of his account to this date.

| | |
|---|---|
| Dr. to balance on hand at last settlement, - | $183 26 |
| Received since, - - - - - - - | 16 00 |
| Total Received, | $199 26 |
| Paid Janitor per order of Society, - - - | 5 00 |
| Balance on hand, - - - - - - | $194 26 |

He would also report that two of the gentlemen who were elected last year—Drs. Hermon of Nashville, Tenn., and Evans ot Lexington. Ky., have neglected to pay their initiation fee, so that according to the by-laws, they cannot be considered as members.

Drs. Bonsal, Smith, Talbert, and Taft, were appointed to audit the Treasurers account.

CORRESPONDING SECRETARY'S REPORT.

The Corresponding Secretary would respectfully report that in accordance with his instructions, the resolutions in regard to the prize essay, were forwarded to and published in the *American Journal of Dental Science;* The *Dental News Letter;* The *Dental Recorder*, and *Dental Register of the West.*

GEO. WATT, *Cor. Sec'y.*

The auditing committee reported that "They found upon examination, the report of the Treasurer of the M. V. A. correct."

H. R. SMITH,<br>H. S. TALBERT,<br>J. TAFT. } Committee.

Your Committee appointed to have 500 copies of the constitution printed, submitted the following report.

Your committee find the constitution needs further emendations, they therefore deferred printing until the Society could act.

A. M. LESLIE.<br>C. BONSAL. } Committtee.

The Committee appointed to examine and report on the Essays presented for the prize of $100, by this Society, respectfully report that three Essays have been presented for their examination, which they have read and of which they present the following:—Two of these Essays, strictly speaking, fail to meet the requirement of fifty pages duodecimo. Yet they can not make this a special objection to them, for they would probably make that number of pages, put up in large type, and they feel that the merit of such articles depend more on brevity than length. It appears by the resolutions of the Society, that the Committee were required to approve and have the best Essay printed, that the Association may read it also. Yet they were not furnished with the necessary funds for that purpose. This, however, has really not prevented the printing of such as they might have selected; but they have not had the time to make the necessary and careful examinations and get the same printed.

One of these Essays the Committee would like to have the Society examine. If the Society sees proper, it can have it printed for the more careful perusal of the Association, and thus get their ultimate action on it. The Committee think that haste in this matter is to be avoided. The reputation of the Society requires that an article which they *endorse* should be such as to commend itself to the profession and public.

The Essay which they would like to have the Society examine, possesses many excellent points, and is well written, and if it met in a direct manner more especially the prejudices of the community—would be just such as we should desire.—We think that a popular treatise should be directed to this special object, viz., the correcting the false impressions existing in the community in relation to Dental operations. We regret that the original resolutions of the Society in relation to Essays should not have been more explicit.

JAMES TAYLOR,

J. TAFT,

W. H. GODDARD,

A. M. LESLIE.

} Committee.

The Committee to confer with the College Association reported as follows:

The Committee to arrange with the Building Committee of the College Association, respectfully report, that by arrangements made, space for a cabinet case has been obtained in the cabinet room of the College, and the Faculty of the College agree to afford this and the other conveniencies required for the Society's meetings on such pecuniary consideration as the Society may deem proper. Your Committee reccommend that a suitable cabinet be procured and placed in the allotted space.

A. M. LESLIE,

C. BONSALL,

J. TAYLOR.

} Committee.

The Examining Committee reported the name of Dr. C. H. Quinlan, of Chicago, for membership. He was unanimously elected by ballot.

Microscopic Committee reported the following: Your Com mittee on Microscopic observations, would report, that owing to difficulties which they could not control or overcome in the time allotted to them, they have found it impossible to carry out the design of the appointment, and would respectfully ask further time.

J. TAFT,

JAS. TAYLOR, } Committee.

A. M. LESLIE.

Report accepted and time granted.

Dr. Talbert moved that when the meeting adjourns it continues adjourned until $3\frac{1}{2}$ o'clock, P. M., and that the first business then be the President's Address. Adopted.

After some discussion touching the report of the Committee on Essays, Dr. Smith moved, that the reading of the Essay therein recommended for examination be the first business of the session in the following morning. Carried.

Dr. Taft moved, that members of the profession not members of the Association, be, and are hereby invited, to take part in the discussions if they see proper. Adopted. Adjourned.

AFTERNOON SESSION.

The Minutes of the Morning Session were read and approved.

The President delivered the opening address. Subject, "Association."

Dr. Taft moved, that the thanks of the Association be presented to Dr. Goddard for the able address just delivered, and that a copy be requested for publication. Adopted.

The report on Dental Progress was read by Dr. Leslie, so far as he had time to prepare it. He also presented several specimens, illustrative of improvement; when, on motion, the further reading of the report was deferred until a future session.

Adjourned until 9 A. M., the following day.

SECOND DAY, MORNING SESSION.

The minutes of previous session read and approved.

The Examining Committee presented the names of Henry Munroe, of Lebanon, Ky., and Jonathan Douthett of Xenia Ohio, for membership. Ballot had and gentlemen elected.

On motion, the Committee on Essays was instructed to report the one which they may think best of those presented. The Committee retired and brought in a report, which, on motion, was accepted.

In accordance with instructions of the Association, the Committee on Essays respectfully report that the Essay marked No. 3, and presented by "John Gilpin," is in their opinion the best presented, and recommend that the prize promised of $100, be awarded accordingly.

JAMES TAYLOR,

WM. H. GODDARD,

A. M. LESLIE,

J. TAFT.

} Committee.

The Essay was then read, and on motion, the recommendation of the Committee, awarding the prize ($100) to John Gilpin, author of the best Essay, was adopted.

On motion, the envelope containing the true name of the author was opened by the President, when the card of George Watt, D. D. S., M. D., of Xenia, Ohio, was found therein.

On motion, it was resolved, that a Committee be appointed to make such amendments in the Essay—just read—as may seem necessary, to meet the demands of the profession, and that the author be one of the Committee.

On motion, resolved, that the Chair appoint the Committee. Chair appointed Drs. Watt, Taylor and Taft.

Dr. Goddard presented the following resolution. Whereas, the American Society of Dental Surgeons will hold their next meeting in this city, on the 8th day of May next. Therefore, *Resolved*, That we deem it expedient that a called meeting of this Association be held at that time, and the President is hereby authorized to call such meeting. *Resolved*, That we cordially invite all members of the profession—not members of this Society—to be present, and co-operate with us on the above occasion, that we may be able to extend a hearty welcome and the right hand of fellowship to our Eastern brethren. Adopted. Adjourned to 2½ P. M.

Afternoon Session. Minutes Approved.

Dr. Talbert read an Essay on the preparation of cavities for filling.

On motion of Dr. Watt, resolved, that the thanks of the Association be tendered to Dr. Talbert, and that a copy of his address be requested for publication.

Dr. Smith was granted further time for the preparation of an Essay.

Dr. Watt read an Essay on alloys of metals.

Dr. Ulrey moved that the thanks of the Association be tendered to Dr. Watt, and that his Essay be requested for publication. Carried.

Dr. Hamill asked further time for the preparation of his address.

Dr. Ulrey asked to be excused from the consideration of atmospheric plates, which was granted, and he was requested to prepare an Essay for our next meeting (annual.)

A motion was made, that the Chair appoint a Committee to examine extracting instruments, presented in competition for the prize offered last year by the Society. Carried.

The Chair appointed Drs. Taylor, Talbert and Webster.

Dr. Leslie moved, a Committee consisting of three be appointed to examine the Teeth sent on by Jones, White & Co., and report such action as they may deem proper. The Chair appointed Drs. Hamill, Taft and Ulrey.

Dr. Bonsall moved the thanks of the Association to Dr. Peebles for his scotch-stone holder.

On motion, resolved, that the business for the morning session, at 9 o'clock, be the election of officers.

The Committee on Forceps reported, and report accepted.

The Committee appointed to examine and report concerning extracting instruments, respectfully report that but one set was placed in their hands. These were presented by Messrs. Sherwood & Brown, and that their adaptation to the teeth, and variety, meeting the wants of the profession in almost all cases, also, finish and style is such that they recommend them for the award offered.

JAMES TAYLOR, A. S. TALBERT, W. R. WEBSTER. } Committee.

On motion, report of Committee adopted.

*Resolved*, That an order be drawn on the Treasurer, in favor of Messrs. Sherwood & Brown, in accordance with the above.

Mr. Oram, of the firm of Oram & Armstrong, of Philadelphia, presented a card of Gum Teeth for examination of the Society. Referred to Committee on porcelain teeth.

Society resumed the regular order of business, discussed Nos. 1 and 2 of miscellaneous bussiness. Adjourned to 7½ o'clock, P. M.

EVENING SESSION met and discussed Nos. 3, 4, and 5 of miscellaneous business.

The following report on Porcelain Teeth was submitted by Committee.

Gentlemen of the M. V. A. of D. S. Your Committee on Artificial Teeth, would respectfully report, that the specimens presented by Messrs. Jones, White & McCurdy, are worthy our highest commendation. Every variety of shape, size and color was represented, and we are happy to say that they fully sustain the justly high reputation they already enjoy, among the members of the profession.

We consider those presented to the Miss. Valley Association a valuable acquisition to its cabinet—for which they have our warmest thanks.

Specimens were also examined from the manufactory of Messrs. Oram & Armstrong; these were all Gum Teeth, and presented a beautiful appearance, and we recommend them to the favorable consideration of the profession.

J. TAFT, J. G. HAMILL, J. P. ULREY. } Committee.

Report accepted and adopted.

*Resolved*, That the business at to-morrows' session, 9½ o'clock

A. M., be election of officers. Adjourned to 8 o'clock A. M.

THIRD DAY. Minutes of previous session, read and approved.

Dr. Goddard moved, that the thanks of the Association be, and are hereby rendered to Messrs. Watt, Sherwood and Brown, for the entertainment last evening. Carried.

Society discussed Nos. 6, 7 and 8 of miscellaneous business.

Election of officers followed, with following result:

*President.*—A. S. TALBERT.

*Vice President.*—J. G. HAMILL.

*Rec. Secretary.*—A. M. LESLIE.

*Cor. Secretary.*—GEO. WATT.

*Treasurer.*—C. BONSALL.

*Executive Com.*—Drs. Smith, Ulrey and Douthet.

*Examining Com.*—Drs. Webster, Taft & Collins.

*Com. on Den. Progress.*—Drs. Watt, Leslie & Smith.

The following gentlemen, in addition to those continued over, were appointed to prepare Essays for next annual meeting, W. S. Howe and E. Collins.

The Committee on Clasps was discharged.

The Committee on Printing the Constitution granted further time.

The President resigned the Chair in favor of the President elect—Dr. Talbert.

It was moved, the thanks of the Society be tendered to Dr. Goddard, for the able manner he has presided.

On motion of Dr. Taylor, resolved, that the Committee on Dental Progress be requested to continue the report on the subject; embodying all the new and important improvements which have been made for two years past, and ensuing year, and arrange the same for publication by the next annual meeting.

On motion of Dr. Webster, resolved, that the thanks of the Association be rendered to Dr. Hunter for his exhibition of specimens, showing the progress made by him in continuous gum work, also, for the communication accompanying the same; and that the Association heartily wish him the success he richly merits.

On motion of Dr. Douthett, resolved, that the Association request Dr. Taylor—if he deems it practicable—to enlarge the Dental Register, and that we hereby pledge our *influence* to continue and extend its circulation.

On motion of Dr. Taylor, resolved, that this Association regard the conviction of Dr. Beale, on the evidence which was presented, as a bad and dangerous precedent, endangering the character and reputation of members of the profession.

Moved an order be drawn on the Treasurer for five dollars, in favor of John Reese, the acting Janitor, for services done the Society.

Dr. Goddard moved, that members send in specimens. Carried.

Dr. Goddard moved, that Dr. Bonsall be appointed to make arrangements for use of rooms for American Society; also, to arrange with the Faculty the cost of the accommodations offered this Society. Carried.

Dr. Bonsall moved, the Secretary be requested to prepare a copy of the minutes in time for publication in the April No. of the Dental Register. Carried.

Minutes read and approved.

On motion, adjourned, to meet again at Dental College, February 20th, 1856, at 10 o'clock A. M.

---

### MINUTES OF THE THIRD ANNUAL MEETING OF THE OHIO DENTAL COLLEGE ASSOCIATION.

The Association met according to adjournment at 2 P. M., of Monday, Feb. 19, 1855, in the New Ohio Dental College buildings.

Present, Drs. Taylor, Taft, H. R. Smith, Duncan, J. M. Brown and Bonsall.

The minutes of last year were first read, and then the building committee handed in the different propositions they had received for the building, and gave some account of their la-

